# Live attenuated enterotoxigenic *Escherichia coli* (ETEC) vaccine with dmLT adjuvant protects human volunteers against virulent experimental ETEC challenge

**DOI:** 10.1016/j.vaccine.2019.02.025

**Published:** 2019-03-28

**Authors:** Clayton Harro, A. Louis Bourgeois, David Sack, Richard Walker, Barbara DeNearing, Jessica Brubaker, Nicole Maier, Alan Fix, Len Dally, Subhra Chakraborty, John D. Clements, Ingelise Saunders, Michael J. Darsley

**Affiliations:** a Center for Immunization Research, Johns Hopkins Bloomberg School of Public Health, Baltimore, MD, USA; b PATH, Washington, DC, USA; c The Emmes Corporation, Rockville, MD, USA; d Tulane University, School of Medicine, New Orleans, LA, USA; e TDVaccines, Odense, Denmark; f MD Biologic Ltd., Cambridge, United Kingdom

## Abstract

**Background:**

There is no licensed vaccine against enterotoxigenic *Escherichia coli* (ETEC), a major cause of diarrhea-associated morbidity and mortality among infants and children in low-income countries and travelers. The results of this vaccination/challenge study demonstrate strong protection by an attenuated ETEC vaccine candidate, ACE527, when co-administered with a mucosal adjuvant, the double-mutant heat-labile toxin (dmLT) of ETEC.

**Methods:**

Sixty healthy adults participated in a randomized, placebo-controlled, double-blind study with three doses of lyophilized ACE527 (∼3 × 10^9^ of each strain per dose) administered orally with or without dmLT adjuvant (25 µg/dose). Six months later, 36 of these volunteers and a control group of 21 unvaccinated volunteers were challenged with virulent ETEC strain H10407. The primary outcome was severe diarrhea, defined as passing >800 g of unformed stools during the inpatient period following challenge.

**Findings:**

The vaccine was well tolerated and induced robust immune responses to key antigens. The protective efficacy (PE) against the primary outcome of severe diarrhea was 65.9% (95% confidence interval [CI] 5.4–87.7, p = 0.003). Among subjects receiving the adjuvanted vaccine, the attack rate of severe diarrhea was 23.1, while in unimmunized controls it was 67.7%. The PE against diarrhea of any severity was 58.5% (95% CI 3.8– 82.1, p = 0.016). There was a strong inverse correlation between shedding of the vaccine strain after either of the first two doses and absence of severe diarrhea upon challenge (RR = 0.29, 95% CI 0.08–1.05, p = 0.041). Challenge strain shedding was 10-fold lower in those receiving the adjuvant than in those receiving vaccine alone. The unadjuvanted vaccine was not protective (PE = 23.1%).

**Interpretation:**

The results of this study support further development of ACE527 + dmLT as a vaccine for children in endemic countries and travelers. This is the first clinical demonstration that dmLT can contribute significantly to vaccine efficacy and may warrant testing with other oral vaccines.

(ClinicalTrials.gov registration: NCT01739231).

## 1 Introduction

Morbidity and mortality following diarrhea caused by infection with enterotoxigenic *Escherichia coli* (ETEC) remain a major threat to infants and children living in endemic areas. ETEC is also a major cause of travelers’ diarrhea [1]. In the recent Global Enteric Multicenter Study, ETEC strains producing heat-stable enterotoxin (ST) or both ST and heat-labile toxin (LT) were among the most important pathogens associated with moderate-to-severe diarrhea (MSD) among children younger than 5 years of age in low- to middle-income countries (LMICs) [2]. In that study, children experiencing ETEC-associated MSD were at an increased risk of mortality and stunting. No practical and effective vaccine against ETEC is currently available. The development of a safe and effective ETEC vaccine, a high priority of the World Health Organization [3], may best be achieved by eliciting both antitoxic and anti-fimbrial immunity [1], [5]. Coverage for the B subunit of LT, CFA/I, and coli surface (CS) antigens 1 through 6 should provide coverage against at least 80% of clinical strains [4], [5].

ACE527 is a live, oral, multivalent vaccine comprising three genetically attenuated and engineered strains of ETEC. It contains antigens covering a wide range of ETEC surface colonization factors (CFA/I, CFA/II [CS1, CS2, CS3] and CFA/IV [CS5, CS6]) as well as LT-B, the binding subunit of LT [6], [7], [8]. ACE527 was shown in a Phase 1 trial to be safe, well tolerated and immunogenic in healthy adults at doses of 10^10^ and 10^11^ cfu [9]. These observations were extended in a subsequent Phase 2 vaccination and challenge trial in which two doses of 2 × 10^11^ cfu were administered 21 days apart with subsequent challenge 28 days after the second dose with the highly virulent challenge strain H10407 [10]. The vaccine had a significant impact on diarrhea severity and intestinal colonization by the challenge strain, suggesting the induction of a functional immune response to the CFA/I antigen [10]. Although ACE527 did not demonstrate significant protection against the primary endpoint of MSD (PE = 27%, p = 0.12), vaccinees had a significant reduction of a number of secondary and ad hoc endpoints compared to control volunteers. The vaccine was protective against severe diarrhea (PE = 41%, p = 0.03) defined by the passage of >800 gm of unformed stools during the post-challenge observation period, and vaccine recipients were 2.8 times more likely to pass no unformed stools after challenge compared to placebo recipients (p = 0.04). Among the considerations to improve on these encouraging observations were the inclusion of a third dose in the primary immunization series, as well as the addition of a mucosal adjuvant LTR192G/L211A, also known as the double-mutant heat-labile toxin (dmLT) [12], [13], [14], [15], [16], [17]. The dmLT adjuvant has been shown to be safe in oral doses up to 100 μg [13]. Data are limited on the impact of attenuated LT adjuvants on live attenuated vaccines, but earlier studies with LT(R192G) or mLT by Hartman and colleagues showed that co-administration of mLT with the attenuated EcSf2a-3 *Shigella* vaccine significantly improved its protective efficacy in guinea pigs [17] and adding dmLT to an attenuated *Salmonella*-vectored ETEC vaccine improved its immunogenicity in mice [18].

With the high dose of attenuated cells used in the initial Phase 2b challenge study (∼10^11^ per dose; ∼3 × 10^10^ cfu per strain), a substantial proportion of vaccine recipients experienced gastrointestinal adverse events (AEs) [10]. However, given the modest protection shown by the vaccine [10], additional studies were warranted. Consequently, to further improve tolerability and potentially improve protective efficacy, we moved to a three dose regimen evaluating a lower dose and added dmLT in the current trial.

## 2 Methods

### 2.1 Study design and participants

The intended application for this vaccine is in both travelers to developing countries and infants and children living in LMICs. For both, safety and early indications of efficacy need to be evaluated first in healthy adult volunteers. A three-dose regimen, with doses spaced at four-week intervals, was selected to mimic potential regimens for pediatric applications. Volunteers were recruited for vaccination in Part A from the general population in Baltimore, MD, with eligibility criteria as described previously [9], [10]. In an amendment to the clinical protocol, volunteers who participated in Part A were invited to participate in Part B (challenge phase) approximately six months after completion of the vaccination regimen, while the study was still blinded. A total of 36/60 volunteers from Part A were eligible and agreed to participate in Part B. As the challenge phase followed 6–7 months later, the lower carryover rate of subjects from Part A to Part B was mostly based on availability. These 36 volunteers were re-consented to receive a challenge dose of H10407 ETEC according to the well characterized model [19]. To boost the statistical power of the study, an additional 21 unvaccinated controls were consented to participate in Part B. The control group therefore comprised a mixture of volunteers who had received placebo in Part A (still double-blinded) and new unvaccinated controls (unblinded). The protocol and amendments were reviewed by the Center for Biologics Evaluation and Review under BB-IND#15,181 and reviewed and approved by the Western Institutional Review Board (Olympia, Washington) and by the Johns Hopkins Institutional Biosafety Committee. The use of the H10407 ETEC challenge strain was conducted under BB-IND-12,234.

### 2.2 Randomizaton

The 60 volunteers enrolled for Part A were randomly assigned to one of three treatment groups in the ratio 1:2:2, placebo: ACE527: ACE527 + dmLT. The vaccine doses were provided to the clinical team in blinded containers for administration. The treatment assignment of the volunteers randomized in Part A remained blinded to the clinical team through Part B until database lock. For Part B, 26 volunteers from Part A were eligible and agreed to participate in the challenge phase. Recruitment of Part A participants for Part B was not based on post-vaccination immune status.

### 2.3 Manufacture and delivery of ACE527

Master and Working Cell Banks (WCB) for each of the three vaccine strains that comprise ACE527 were produced under Good Manufacturing Practice at the Walter Reed Army Institute of Research (WRAIR) Pilot BioProduction Facility (PBF), Silver Spring, MD, USA. Vials from the WCBs were expanded into shake flasks to prepare a starter culture which was subsequently transferred into a 30L working volume bioreactor and grown for 5–7 h. At the desired cell density, the cells were harvested by continuous flow centrifugation. The resulting pellet was washed by centrifugation and resuspended in stabilizer solution (100 mM mannitol, 50 mM sucrose), 1.2 mL volumes were aliquoted and lyophilized in 5 mL vials which were stored at −20 °C ± 5 °C. Each strain met pre-set physical, microbiological, biochemical and antigenic criteria before being released for use in the clinical trial.

### 2.4 dmLT

The adjuvant was produced, purified and characterized [11] at the WRAIR PBF. LT(R192G/L211A) was lyophilized in a sodium phosphate buffer supplemented with 5% lactose to give aliquots containing 700 µg of protein in 3 mL vials. The lot of dmLT used in this trial, 1575, met pre-set purity, sterility, biological, antigenic and adjuvant activity criteria before being released for use in this trial.

### 2.5 Vaccine preparation and administration

The vaccine doses for the trial were prepared in the research pharmacy by reconstituting individual vials of each strain and mixing to provide 3 × 10^9^ cfu of each in 10 mL PBS per subject. No more than two hours before dosing, the mixture was added to 190 mL of CeraVacx® buffer (Cera Products Columbia, MD). For the volunteers allocated to receive dmLT, individual vials were reconstituted and diluted to a concentration of 50 µg/mL, from which 0.5 mL was added to the reconstituted vaccine no more than five minutes before dosing. Placebo preparations consisted of 10 mL of PBS mixed with 190 mL of CeraVacx®. The CeraVacx® buffer used as the placebo and to deliver the three vaccine strains in this study has been used for the same purposes in prior Phase 1 and 2b trials of ACE527 [9], [10].

Volunteers were admitted to the inpatient unit of the Center for Immunization Research (CIR) for administration of the first dose and in-patient observation for the three days following that dose. The second and third doses were administered on an out-patient basis 28 and 56 days later. All volunteers were required to fast for 90 min before and after vaccination and were observed closely for 60 min post-vaccination for safety and to ensure no regurgitation of study product. The actual viable dose of each strain in the preparation administered to each cohort was determined by serial dilution and plating of an aliquot of the suspension for Quality Control purposes and confirmation that they were consistent with the intended dose level [∼ total dose of 9x10^9^ cfu; dose variance ranged from 5 × 10^9^ to 1 × 10^10^].

### 2.6 Challenge inoculum preparation and administration

Inocula administered in the challenge phase were prepared from fresh, plate-grown organisms as previously described [19]. The number of CFU in the inocula was determined by dilution plating before and after administration to volunteers.

### 2.7 Safety evaluation and clinical monitoring after vaccination

The primary safety endpoints were: number of serious adverse events; number of AEs leading to withdrawal; number of severe adverse reactions within four weeks of vaccination; number of solicited reactions within one week of vaccination; number of unsolicited AEs within four weeks of vaccination. Solicited AEs included loose stools, diarrhea, nausea, vomiting, abdominal pain, urgency of defecation, malaise, headache, chill, fever, borborygmus (gurgling stomach), anorexia, fever, malaise and headache within one week of vaccination. Evaluations of AEs in the seven days following each dose was via standardized vaccination report cards and monitoring laboratory tests (complete blood counts and clinical chemistry to assess liver and renal status), medical evaluations and targeted physical examinations at the end of the 7-day period. Diarrhea during the period post-vaccination was defined as three or more loose or liquid stools within 24 h; other unformed bowel movements not meeting the definition of diarrhea were recorded as loose stools. Abnormal laboratory values were evaluated for clinical significance by the PI and graded as AEs using U.S. Food and Drug Administration toxicity table guidelines.

### 2.8 Stool microbiology

Stool samples were collected at Days 0, 3, 7, 28, 31, 35, 56, 63 and 84 after the first vaccination for evaluation of vaccine shedding. ACE527 colonies were identified as previously described [9], [10]. After challenge, samples were obtained up to three times each day between challenge and discharge to monitor excretion of the H10407 challenge strain. On the second and fourth days after challenge, quantitative culture was performed to determine the level of H10407, which was expressed as CFU/g stool. When stool samples were not available, a sample was obtained by rectal swab for qualitative assessment of shedding.

### 2.9 Immunogenicity evaluation

Part A of this study aimed to further evaluate safety and immunogenicity of ACE527, as well as to investigate the effect of dmLT on tolerability and immunogenicity of ACE527, however it was not specifically powered to draw conclusions about the effect of dmLT on the level or frequency of immune responses. Using antigens and ELISA methods as previously described in Chakraborty et al. [20], systemic serum IgG and IgA, as well as mucosal IgA responses in the Antibody from Lymphocyte Supernatant (ALS) assay were assessed following vaccination and challenge. Both serum and ALS supernatents were evaluated for antibodies to LTB, CFA/I, CS3 and CS6; but only the serum and ALS responses to LTB and CFA/I will be summarized here since they were considered as the most relevant endpoints to the protective efficacy against challenge with H10407. Serum IgG and IgA responses were measured in all volunteers receiving at least one dose of vaccine or placebo in Part A on days 0, 7, 28, 35, 56, 63 and 84 and were analysed by the level of responses (geometric mean titres) as well as the frequency with which volunteers seroconverted, defined as a 2.5-fold or more increase over the pre-immunization levels [10].

Assessment of IgA secreted by peripheral blood mononuclear cells circulating to the mucosal inductive sites was measured by ALS assay on specimens obtained on the day of and at 3 and 7 days following the first and second immunizations and on the day of and at 7 and 28 days following the third immunization. ALS responses were considered positive if, at any time point after vaccination, they reached 4-fold or more over baseline at Day 0.

ELISA assays of serum or ALS supernatants were performed according to standard protocols using peroxidase-labelled anti-human isotype specific detecting antibodies (KPL, Baltimore, MD). CFA/I was supplied by Dr. E. Oaks at WRAIR and LT-B was purchased from Sigma Aldrich (St. Louis, MO).

### 2.10 Efficacy evaluation

Approximately six months after the last vaccination, volunteers were admitted to the in-patient unit of the CIR and challenged with 2 × 10^7^ cfu of the well characterized ETEC strain H10407. Medical interviews and physical examinations were performed daily by the PI, and additional medical assessments and vital sign measurements were performed by the study team at least three times daily from the day of challenge until discharge. Active surveillance for fever, vomiting, nausea, abdominal pain, abdominal cramping, myalgias, malaise, bloating, flatulence, headache, light headedness, chills, constipation, and anorexia was performed, and severity scored as absent, mild, moderate or severe.

The primary efficacy objective in Part B was to determine the protective efficacy of ACE527 (with or without dmLT) against virulent ETEC challenge. Based on the results of an earlier challenge trial [10] the primary endpoint was severe diarrhea based only on output volume, as defined below.

Each stool was collected in a pre-weighed commercial stool collection hat with lid (FisherScience catelogue no. 025444208), weighed, assessed for the presence of blood and graded as follows: grade 1 (firm, formed), grade 2 (soft, formed), grade 3 (viscous opaque liquid or semi-liquid which assumes the shape of the container), grade 4 (watery, non-viscous, opaque liquid), and grade 5 (clear or translucent, watery or mucoid liquid). For the challenge, diarrhea was defined as the passage of 1 loose stool >300 g, 2 loose stools totaling at least 200 g or 3 loose stools in any continuous 24 h period. This differed from the definition of diarrhea as a solicited AE following vaccination. Severe diarrhea, as the primary endpoint of the challenge, was defined as the passage of 800 g or more of loose or liquid (grade 3–5) stools in all episodes that started within 120 h of challenge. An episode of diarrhea was considered ended if 24 h passed without the passage of further loose stool. If a subject had ongoing diarrhea at 120 h, then the volume of loose stool passed subsequent to 120 h during that episode contributed to the endpoint. Additional diarrhea endpoints were the presence of any diarrhea and the total volume and number of loose stools passed.

### 2.11 Statistical analyses

Clinical and safety data were captured using electronic case report forms. The intention to treat analysis population for post-vaccination immune responses comprised all volunteers who received at least one administration of ACE527 (+/− dmLT) or placebo. The per-protocol analysis population comprised all volunteers who received all three doses of ACE527 (+/− dmLT) or placebo. Immune responses were compared by study arm.

Vaccine efficacy against severe diarrhea and the incidence of any diarrhea was calculated as 1 – (incidence in vaccine + dmLT group)/(incidence in control group) with 95% confidence intervals and were compared by Barnard’s unconditional 1-tailed test for the primary and Fisher’s exact 2-tailed test for the secondary. Barnard’s was used for the primary outcome since it was used for sample size estimation and Fisher’s for the secondary as it is more conservative and there were multiple secondary outcomes. Vaccine efficacy against stool number and weight was calculated as 1 – (median value in vaccine + dmLT group)/(median value in control group) and the outcomes were compared by Poisson Regression for frequency and Wilcoxon 2-sample test for weight. The differences between the ACE527 alone and control groups were not statistically significant and are not reported.

All analyses were performed using SAS® software Version 9.3 or later. All volunteers were evaluated for safety and the immunology results are based on the intention to treat population. Post-vaccination shedding results are based on the number of volunteers with data after each vaccination, and efficacy results are based on all volunteers who participated in the challenge study. A more complete analysis, including analyses based on the per-protocol population, will be presented elsewhere.

## 3 Results

### 3.1 Subject disposition and demographics

**Fig. 1:**
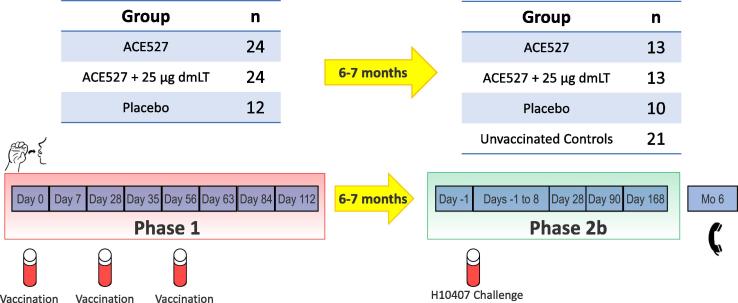
Relationship between trial stages and disposition of study volunteers across parts A and B of the trial. Study design illustrating three doses of test material given in Phase 1, followed 6–7 months later by a Phase 2b challenge study with ETEC strain H10407. Unvaccinated controls were added to achieve appropriate power for determining vaccine efficacy.

**Fig. 2:**
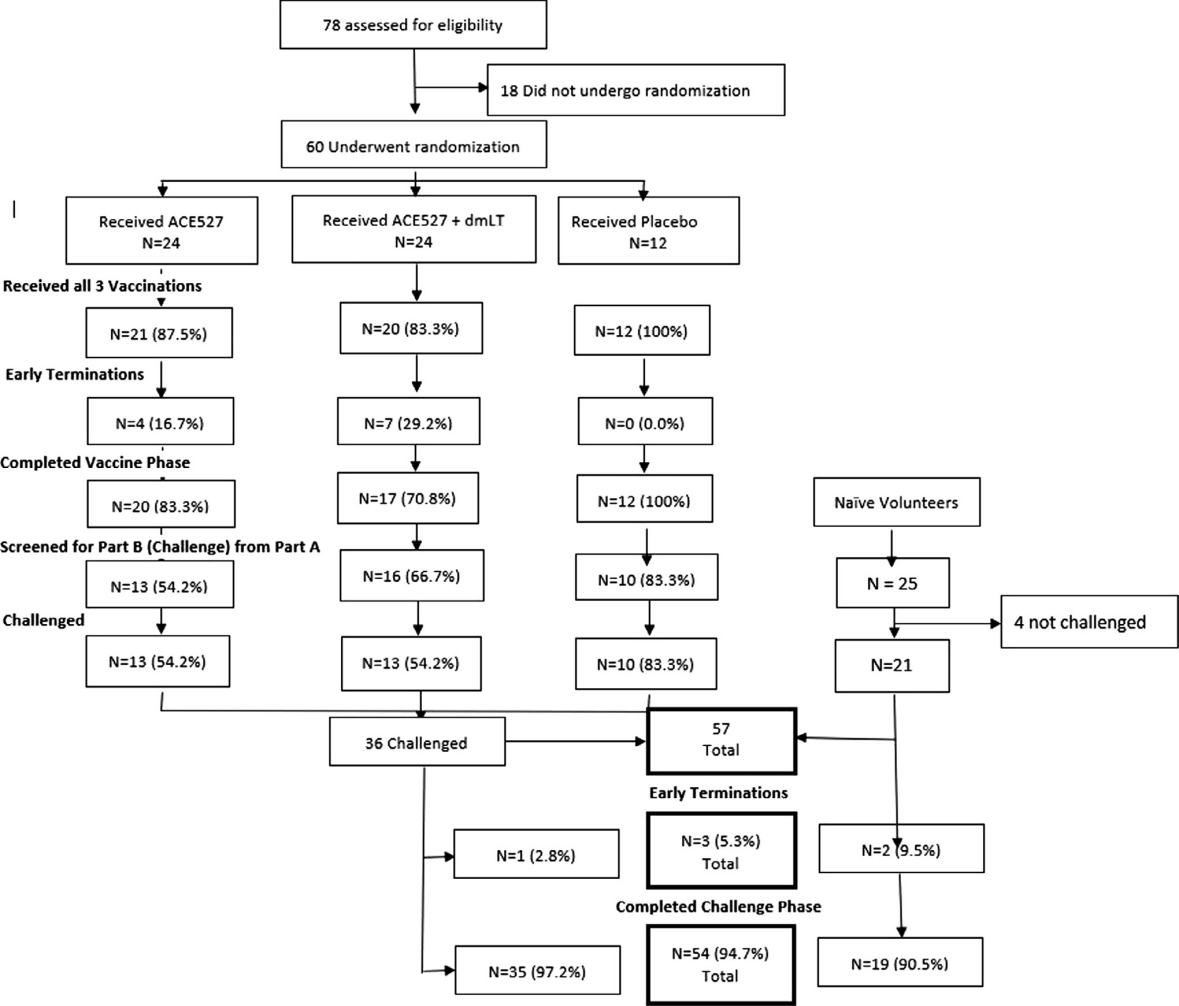
CONSORT Diagram for Study Design.

The relation of the trial stages and the disposition of study volunteers across Parts A and B of the trial are summarized in Fig. 1. In Part A, 78 healthy adult volunteers were screened for eligibility, with 60 enrolled, as illustrated in the CONSORT diagram (Fig. 2). The age range of the enrollees was 19 to 49 years, and the mean age was 36.8 years. The majority of the participants (52 of 60; 86.7%) were Black or African American and 43 (71.7%) were male. The demographics, by study group, are shown in Table 1. A total of 60 volunteers received the first dose of vaccine or placebo, 53 subjects received all three doses of vaccine or placebo and 49 volunteers completed through the final follow-up call on Study Day 98.

**Table 1:** Volunteer demographics by group.

	Part A – Vaccine Phase				Part B – Challenge Phase			
	ACE527	ACE527 + dmLT	Placebo	Total	ACE527	ACE527 + dmLT	Controls	Total
*Total*								
Number of Volunteers	24	24	12	60	13	13	31 (10 from Part A and additional 21)	57

*Gender*								
Female	7 (29.2)	9 (37.5)	1 (8.3)	17 (28.3)	4 (30.8)	6 (46.2)	6 (19.4)	16 (28.1)
Male	17 (70.8)	15 (62.5)	11 (91.7)	43 (71.7)	9 (69.2)	7 (53.8)	25 (80.6)	41 (71.9)

*Race*								
American Indian/Alaskan Native	1 (4.2)	0	0	1 (1.7)	1 (7.7)	0	0	1 (1.8)
Black or African American	19 (79.2)	21 (87.5)	12 (100.0)	52 (86.7)	10 (76.9)	10 (76.9)	29 (93.5)	49 (86.0)
White	3 (12.5)	3 (12.5)	0	6 (10.0)	2 (15.4)	3 (23.1)	2 (6.5)	7 (12.3)

*Age (yrs)*								
Mean	35.8	37.6	37.0	36.8	37.0	37.6	36.5	36.9
Median	36	39	35	37	37	39	36	36
Range	21–48	19–49	26–49	19–49	21–49	27–49	23–49	21–49

*BMI*								
Mean	27.8	26.6	26.0	27.0	28.4	27.9	27.2	27.6
Median	27.0	26.6	25.4	26.6	27.1	27.2	26.5	27.1
Range	21.8–33.8	19.0–33.9	22.1–33.4	19.0–33.9	22.2–35.3	19.1–34.3	21.4–34.9	19.1–35.3

*Number of Vaccinations Received*								
1 Vaccination	1 (4.2)	2 (8.3)	0	3 (5.0)	0	0	0	0
2 Vaccinations	2 (8.3)	2 (8.3)	0	4 (6.7)	0	1 (7.7)	0	1 (1.8)
3 Vaccinations	21 (87.5)	20 (83.3)	12 (100.0)	53 (88.3)	13 (100.0)	12 (92.3)	10 (32.3)	35 (61.4)

### 3.2 Stability of ACE527 strains after lyophilization

On average the vaccine strains lost approximately 0.9 Log of viability during lyophilization and 0.5 Log of viability in the initial three-month period after drying, but lost no further viability at the last point measured, 33 months after manufacture. In addition, the viability of the vaccine batches was assessed independently when each dose was administered. The values obtained on the six dosing days (between 28 and 32 months after manufacture) were close to the expected value (data not shown).

### 3.3 Safety results after vaccination

Although gastrointestinal solicited AEs were somewhat more common among vaccinees than placebo recipients, they were predominantly mild, and there were no significant differences between the groups, neither overall (p = 0.10) nor for any specific event (Table 2). The most commonly reported event was loose stools (18/60 [30.0%] among all groups), which were reported for 5/24 (20.8%), 11/24 (45.8%) and 2/12 (16.7%) of those receiving ACE527, ACE527 + dmLT and placebo, respectively, with all events assessed as mild. The next most commonly reported events were borborgymus (14/60 [23.2%], overall), abdominal pain (12/60 [20.0%], overall) and urgency of defecation (11/60 [18.3%], overall), without statistically significant differences across arms. Nausea and vomiting were reported for a small number of participants: in less than 20% overall and in each of the treatment arms, and rising to the level of moderate in no more than a single participant in each arm.

**Table 2:** Part A: Proportion of Volunteers with Any Solicited Event.

Reactogenicity	ACE527 n = 24		ACE527 + dmLT n = 24		Placebo n = 12		Fisher's Exact Test Moderate/Severe	Fisher’s Exact Test Any Severity
	Any Severity n (%)	Moderate/Severe n (%)	Any Severity n (%)	Moderate/Severe n (%)	Any Severity n (%)	Moderate/Severe n (%)	P-Value	
Fever	1 (4.2)	0 (0.0)	1 (4.2)	1 (4.2)	0 (0.0)	0 (0.0)	1.0000	1.0000
Nausea	4 (16.7)	0 (0.0)	3 (4.2)	1 (4.2)	0 (0.0)	0 (0.0)	1.0000	0.6610
Vomiting	2 (8.3)	1 (4.2)	1 (4.2)	1 (4.2)	1 (8.3)	0 (0.0)	1.0000	1.0000
Abdominal Pain	3 (12.5)	1 (4.2)	7 (29.2)	3 (12.5)	2 (16.7)	0 (0.0)	0.5178	0.4936
Urgency of Defecation	4 (16.7)	1 (4.2)	6 (25.0)	3 (12.5)	1 (8.3)	1 (8.3)	0.8326	0.9665
Boborgymus (gurgling)	6 (25.0)	1 (4.2)	6 (25.0)	1 (4.2)	2 (16.7)	0 (0.0)	1.0000	0.9665
Malaise	1 (4.2)	1 (4.2)	2 (8.3)	0 (0.0)	1 (8.3)	1 (8.3)	0.6746	1.0000
Headache	5 (20.8)	3 (12.5)	7 (29.2)	4 (16.7)	1 (8.3)	0 (0.0)	0.4995	0.8553
Anorexia	3 (12.5)	2 (8.3)	4 (16.7)	0 (0.0)	1 (8.3)	1 (8.3)	0.4109	1.0000
Chills	0 (0.0)	0 (0.0)	1 (4.2)	0 (0.0)	1 (8.3)	0 (0.0)	1.0000	0.3487
Loose stools	5 (20.8)	0 (0.0)	11 (45.8)	0 (0.0)	2 (16.7)	0 (0.0)	1.0000	0.6766
Diarrhea	1 (4.2)	1 (4.2)	1 (4.2)	1 (4.2)	0 (0.0)	0 (0.0)	1.0000	1.0000
Temperature	0 (0.0)	0 (0.0)	0 (0.0)	0 (0.0)	0 (0.0)	0 (0.0)	1.0000	1.0000

Similarly, there was no difference in reporting of unsolicited AEs across study arms, overall (data not shown). The only individual category of events for which there was a difference between groups was for gastrointestinal disorders, with a significant difference between those receiving ACE527 + dmLT and those receiving placebo. This difference was due to higher rates of flatulence among those receiving ACE527 + dmLT, reported for 4.2% of those receiving vaccine alone, 33.3% of those receiving ACE527 + dmLT and for no placebo recipients (overall p-value across groups of 0.006, 0.024 for the comparison of vaccine with dmLT to placebo, and 0.023 for the comparison of vaccine with dmLT to vaccine alone).

### 3.4 Vaccine shedding results

Shedding was defined as detection of any of the bacterial strains at any time after the first, second or third dose of vaccine. ACE527 strains were recovered in the stool of the majority of volunteers following vaccination, consistent with observations in previous studies [9], [10]. Ninety-two percent of the volunteers (22/24) in the ACE527 group and 83% of the volunteers in the ACE527 + dmLT group (20/24) shed a vaccine strain for at least one day (data not shown). Shedding of ACE527 occurred significantly more after the first vaccination in volunteers who received ACE527 alone compared with volunteers who received ACE527 + dmLT: 22/24 (91.7%) versus 16/24 (66.7%), respectively, on day 3 (p = 0.033) and 11/24 (45.8%) versus 3/24 (12.5%), respectively, on day 7 (p = 0.011) based on the Chi-Square test. Data are summarized in Table 3. The number of volunteers shedding ACE527 declined with the second and third doses, and the differences between the groups vaccinated with or without dmLT were not statistically significant. Among the three vaccine strains, the ACAM 2027 strain was the most effective colonizer, with 71% and 54% of the volunteers in the ACE527 alone and ACE527 + dmLT groups shedding this strain, respectively (data not shown).

**Table 3:** Shedding of the ACE 527 vaccine over the course of the 3-dose primary immunization series.

	Vaccination 1				Vaccination 2				Vaccination 3			
Days post-vaccination		0	3	7		0	3	7		0	3	7
	N	n (%)	n (%)	n (%)	N	n (%)	n (%)	n (%)	N	n (%)	n (%)	n (%)
ACE527	24	0	22	11	23	1	14	5	21	0	3	1
		(0.0)	(91.7)	(45.8)		(4.3)	(60.9)	(21.7)		(0.0)	(14.3)	(4.8)
ACE527 + dmLT	24	0	16	3	22	0	12	3	20	0	4	0
		(0.0)	(66.7)	(12.5)		(0.0)	(54.5)	(13.6)		(0.0)	(20.0)	(0.0)

No placebo recipients shed the ACE527 vaccine.

### 3.5 Immunogenicity results after vaccination

The rates of response in serum and ALS to LTB and CFA/I are shown in Table 4. Consistent with previous results [10], the strongest serum responses were observed against LT-B. In this study, ALS responses to LTB and CFA/I were comparable; 71% of volunteers receiving ACE527 alone had a 4-fold increase or more in ALS response rate compared to 88% in the group where dmLT was co-administered (p = 0.287). The response rate among controls was 0%. The serum responses to LT-B were not significantly different between the two groups, with combined response rates in the two ACE527 groups of 72.9% IgG and 27.1% IgA. Corresponding response rates for CFA/I were also not significantly different between the two ACE527 groups, with combined response rates of 69% ALS, 47.9% for serum IgA and 22.9% serum IgG. A more complete analysis of mucosal and serum antibody responses to other key colonization factor antigens in the vaccine as well as those following the challenge with H10407 will be presented elsewhere. These studies will include an assessment of B-cell memory, antibodity avidity, and a more in depth analysis of the antigenic breadth of the mucosal antibody response using proteomic microarrays. The LTB and CFA/I responses are highlighted here since they were believed to be the most relevant in the context of the H10407 challenge that followed. It is important to note that the frequency of mucosal and serum immune responses to CFA/I and LTB were not significantly different among all immunized volunteers in Part A of the study and the subset of volunteers that went on to be challenged with H10407 in Part B of the study.

**Table 4:** Frequency of serum IgG and IgA and ALS IgA responses at any time prior to challenge, by dosing group.

Antibody	Group	No. of responders (%)^a^	
		All vaccinees Part A (N = 12, 24, 24)^b^	Part A volunteers challenged in Part B (N = 10, 13, 13)^b^
*Anti-LTB*			
ALS IgA	Placebo	0	0
	ACE527 alone	17 (70.8)	9 (69.2)
	ACE527 + dmLT	21 (87.5)	10 (76.9)
Serum IgA	Placebo	0	0
	ACE527 alone	4 (16.7)	2 (15.4)
	ACE527 + dmLT	9 (37.5)	6 (46.2)
Serum IgG	Placebo	0	0
	ACE527 alone	17 (70.8)	10 (76.9)
	ACE527 + dmLT	18 (75.0)	11 (84.6)

*Anti-CFA/I*			
ALS IgA	Placebo	0	0
	ACE527 alone	20 (83.3)	11 (84.6)
	ACE527 + dmLT	13 (54.2)	9 (69.2)
Serum IgA	Placebo	0	0
	ACE527 alone	14 (58.3)	6 (46.2)
	ACE527 + dmLT	9 (37.5)	6 (46.2)
Serum IgG	Placebo	2 (16.7)	1 (10.0)
	ACE527 alone	6 (25.0)	5 (38.5)
	ACE527 + dmLT	5 (20.8)	3 (23.1)

a Threshold = 2.5x for serum responses, 4x for ALS.

b Of the 24 volunteers in each active group and 12 placebo volunteers in Part A, 13 and 10 respectively were challenged in Part B. The frequency of mucosal and serum antibody responses to CFA/I and LTB in the Part A volunteers and the Part B subset are not statistically significantly different.

### 3.6 Efficacy results

The challenge phase (Part B) included 13/24 who had been vaccinated with three doses of ACE527 alone, 13/24 vaccinated with ACE527 + dmLT (12 of these had received three doses while one had received only two doses), 10/12 vaccinated with three doses of placebo in Part A and an additional 21 unvaccinated volunteers. The demographics of those volunteers who received the challenge are shown in Table 1, and the immune responses post-vaccination for those who participated in Part A are shown in Table 4. The demographic characteristics and the immune response frequencies of the challenged subset were comparable to the larger overall study group. The attack rate for the primary study endpoint in Part B was comparable between the 10 blinded control volunteers from Part A (70%) and the 21 unvaccinated controls added for Part B (66.7%). There was no significant difference in attack rates in the placebo recipients from Part A and the additional control volunteers (see Material and Methods).

**Fig. 3:**
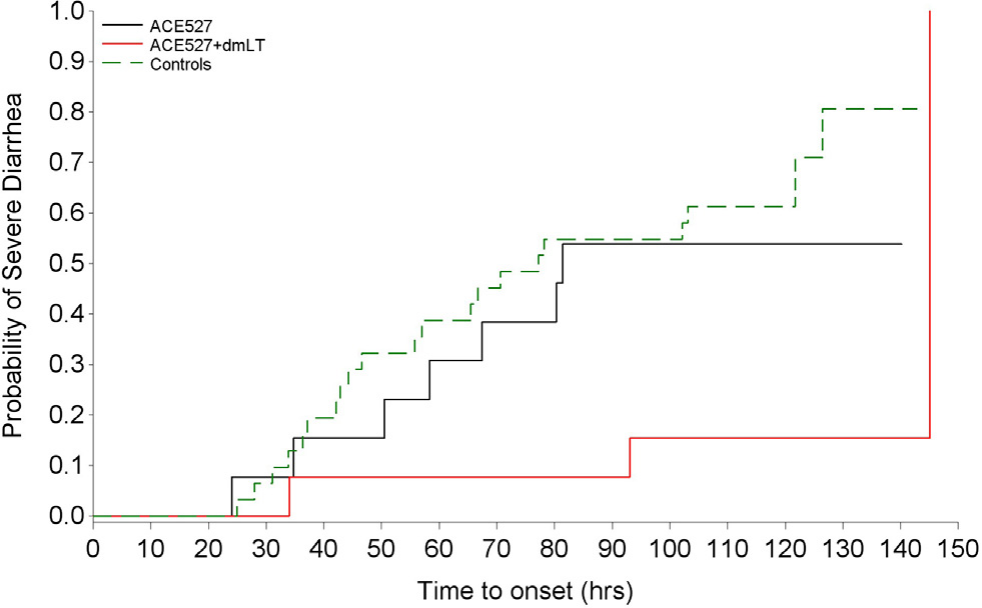
Delay of time to reach the primary severe diarrhea endpoint in volunteers given ACE527 vaccine with or without dmLT. Subjects who did not meet the primary endpoint were censored at the maximum of (a) 120 h, (b) 24 h from the time of last 3–5 grade stool passed in the 120 h time period, and (c) end of the last diarrheal episode that began within the 120 h time period. Log-ranked tests were used to evaluate differences between groups: ACE527 vs. ACE527 with dmLT (p = 0.0387), ACE527 with dmLT vs. Controls (p = 0.0046), ACE527 vs. Controls (p = 0.3715).

The efficacy outcomes are summarized in Table 5. The incidence of severe diarrhea following challenge in the control group was 67.7%, consistent with the attack rate seen in previous studies using this model [10]. In the ACE527 alone group, the attack rate for severe diarrhea was slightly reduced to 53.8% (corresponding to a non-significant protective efficacy of 20.5%), but in the ACE527 + dmLT group the attack rate fell to 23.1%, a protective efficacy of 65.9% (95% CI 5.4 to 87.7%; p = 0.003). In the ACE527 + dmLT group, there were also highly significant reductions in the incidence of diarrhea of any severity (VE = 58.5%, p = 0.016 2-sided FET) and in the median total weight and total number of stools passed. Further, there was a 10-fold reduction in shedding of the H10407 challenge at day 2 post-challenge in the protected dmLT group vs. vaccine only and control groups. The median shedding level in controls at day 2 was 4.9 × 10^7^ cfu/gm of stool while in volunteers given the dmLT-adjuvanted vaccine the median shedding level was 3.7 × 10^6^. In addition, based on Kaplan-Meir analysis, the few volunteers in the ACE527 + dmLT group that met the primary severe diarrhea endpoint did so much later than control volunteers (p = 0.005) or volunteers receiving ACE527 alone (p-value < 0.04) (Fig. 3).

**Table 5:** Efficacy endpoints.

	ACE527/dmLT	ACE527	Controls	Vaccine Efficacy of ACE527 + dmLT	
	N = 13	N = 13	N = 31	% 95% CI	P value
*No. volunteers with Severe Diarrhea: Primary Endpoint*					
	3 (23.1%)	7 (53.8%)	21 (67.7%)	65.9% (5.4 to 87.7)	0.003*
*No. volunteers with Diarrhea of any severity*					
	4 (30.8%)	10 (76.9%)	23 (74.2%)	58.5% (3.8 to 82.1)	0.016$
*Median total weight of loose stools after challenge (120 h obs)*					
	30 g	859 g	1347 g	97.8%	0.008
*Median total number of loose stools after challenge (120 h obs)*					
	1	13	10	90.0%	0.036

* Barnard’s 1-tailed test (was 0.008 by FET 1-tailed).

$ Fisher’s exact 2-tailed test.

Shedding of the three different vaccine strains (ACAM 2022, 2025 and 2027) [8] was evaluated for a potential relationship with positive outcomes post-challenge as measured by diarrhea of any severity. A strong relationship was seen between shedding of ACAM 2025 (expresses the CFA/I colonization factor also expressed by the H10407 challenge strain) and resistance to subsequent challenge. Eight out of 10 volunteers (80%) shedding ACAM 2025 did not show any disease, whereas among the 16 non-shedders only 5 (31%) did not get diarrhea in the absence of shedding (X^2^ = 5.63; p = 0.04 two-tailed test). As expected, no association with shedding and protection was seen with the non-CFA/I expressing strains in the vaccine.

In summary, vaccination with ACE527 induced significant LT-B and CFA/I responses, comparable to those seen in previous trials. There were no significant differences between the vaccine alone and vaccine-adjuvanted groups with respect to the frequency and magnitude (data not shown) of responses to the two antigens. Responses to LT-B tended to be higher in dmLT recipients whereas responses to CFA/I tended to be lower in dmLT recipients. Among the 36 randomized volunteers from Part A that went on to be challenged with H10407 in Part B, there was a strong trend toward those having modest anti-CFA/I ALS responses (≥3 fold over baseline) at some point post-immunization having a reduced risk of developing severe diarrhea post-challenge (PE = 43.8%; p = 0.09 by Fishers Exact Test) (data not shown). Anti-LTB ALS responses did not show a similar trend. It is unclear if the substantially longer interval between the primary immunization series and challenge in the present study (6–7 months) contributed to the diminished association between anti-CFA/I and LTB ALS responses and protection seem in this study.

## 4 Discussion

ACE527 is the first ETEC vaccine containing all necessary cellular components for broad colonization factor and LT-toxin coverage (complete vaccine) to demonstrate protection in humans following a challenge. Further, this trial provides the first indication that dmLT can enhance the protective efficacy of an orally delivered vaccine. These data are similar to earlier results obtained with the vaccine given at a log higher dose without dmLT. The dose-sparing effect of dmLT [12], [14] appears to have compensated for the reduction in vaccine dose and may account for why higher levels of protection were seen 6–7 months post-immunization in contrast to the weaker protection (PE = 41%) seen in the earlier study at 4–6 weeks post-immunization [10].

In general, ACE527, with or without dmLT, appeared to be better tolerated in this study than previously, in which the vaccine dose was a log higher. Notably, in the previous study, 7 (19.4%) participants reported vomiting, compared to 2 (8.3%) and 1 (4.2%) receiving ACE527 and ACE527 + dmLT, respectively, in the current study. In both studies, no placebo recipients reported vomiting. Further, in the previous study, the majority of episodes of vomiting were moderate or severe, whereas in this study, only one participant in each vaccine group experienced vomiting that was greater than mild. Similarly, in the previous study, 11 (30.6%) participants reported nausea, whereas in this study, nausea was reported by only 4 (16.7%) and 1 (4.2%) of the participants receiving ACE527 and ACE527 + dmLT, respectively. In addition, in the previous study, 6 (16.7%) participants reported diarrhea, whereas in this study, 1 (4.2%) participant reported diarrhea in each of the two groups receiving vaccine. Although the pattern of solicited AEs in this study was consistent with that reported previously [10], with the majority being gastrointestinal, these events were reported less often, possibly due to the lower dose. The dose-sparing effect of dmLT [12], [14] might allow for further dose reduction and increased tolerability without reduction in protection. The potential for further dose reduction is particularly important for progression to studies in young children and infants. Previous studies with inactivated whole cell ETEC in Bangladeshi children and infants showed the value of fractional dosing in improving vaccine tolerability [23]. Using lower doses would also serve to reduce vaccine cost. The protection observed in this stringent challenge model suggests considerable potential for protection against naturally occurring infections, which may present considerably less of a challenge than that from 2 × 10^7^ cfu dose used in the present study.

Despite the small number of volunteers in each active vaccine group, limiting precision of the results, the protection observed for ACE527 + dmLT was statistically significant. Further studies will be required to verify that these results are reproducible, and a larger study would provide a more precise and reliable assessment of protection. The high level of protection observed in this study strongly supports further development of suitable formulations for trials in descending-age groups in countries where ETEC is endemic and a major cause of infant mortality and morbidity.

Shedding reduction seen two days following challenge may be an important factor in protection. In the previous challenge trial using 10^11^ cfu of vaccine, a significant drop in shedding upon challenge was observed. The present study likewise demonstrated a log reduction in shedding. Of note, in this study, the protective response was seen 6–7 months after immunization, suggesting a rapid anamnestic response a relatively long time after vaccination. The observed shedding of the CFA/I positive ACAM2025 vaccine strain after immunization and the vaccine-induced anti-CFA/I IgA ALS response (≥3-fold increase over baseline) suggest a role for ant-CFA/I immunity as a marker for longer term protection against H10407 challenge. Field studies from Egypt indicate that anti-CFA/I immunity can be associated with a reduced risk of illness from CFA/I-expressing ETEC [21].

This study indicates that protective immune responses may be relatively long-lived. The value of dmLT in enhancing and extending the protective efficacy of ACE527 is further highlighted by the fact that the two-dose ACE527 regimens evaluated in the prior Phase 2b immunization and challenge study was only 41% efficacious against the severe diarrhea endpoint as defined in this study, even though the H10407 challenge was delivered much sooner after vaccination in that study, 4–6 weeks after the primary immunization. These data indicate that the use of dmLT may have enhanced vaccine-induced immunological memory. Immunological data obtained to date in this trial do not provide insight into the dmLT’s mechanism of protection. However, we also cannot rule out the impact of dmLT on T-cell responses [24], [25].

The immunization dose used here was a log less than the dose used in a previous challenge study involving ACE527 [10]. The third dose of vaccine alone did not contribute to meaningful protection, and the study design cannot address whether the third dose was necessary for the substantial beneficial effect observed when dmLT was co-administered. It has been shown for killed-cell vaccines that three doses of antigen are necessary to promote antigen-driven expansion of high-affinity IgA B cells being distributed to and expanded in the germinal centers in multiple Peyer’s patches [22]. ACE527 is live, but it is possible that the three-dose phenomenon reported for killed cells could also apply to some live attenuated cells.

## 5 Conclusion

In this study, the addition of dmLT adjuvant to three vaccine doses at 10^10^ cfu induced a strongly protective immune response in a clinical challenge study, which was not seen with vaccine alone. Compared to the earlier Phase1 and 2b studies using the higher vaccine dose, the lower dose was better-tolerated and the principle AEs of diarrhea and vomiting were substantially reduced, even with the addition of dmLT adjuvant. This is the first time that a complete vaccine has shown such convincing protection against challenge with the virulent ETEC strain H10407 in a controlled human infection model, a fact made more significant by the gap of approximately six months between vaccination and challenge. The immune mechanisms behind this protection remain to be elucidated, but the contributing role of the dmLT adjuvant to vaccine efficacy is clearly shown by the data presented here. The successful immune protection by the lower vaccine dose levels in combination with dmLT adjuvant strongly argue for a descending-age field evaluation to demonstrate safety and immunogenicity of this vaccine construct in the target population, infants and children in LMICs.
